# Trajectory of Smoking and Incidence of Atherosclerotic Cardiovascular Disease among Korean Young Adult Men

**DOI:** 10.3390/ijerph16122219

**Published:** 2019-06-24

**Authors:** Yongho Jee, Jooeun Jeon, Joung Hwan Back, Mikyung Ryu, Sung-il Cho

**Affiliations:** 1 Department of Public Health Science, Graduate School of Public Health, Seoul National University, 1 Gwanak-ro, Gwanak-gu, Seoul 08826, Korea; jyongho@snu.ac.kr; 2 Institute for Health Promotion, Graduate School of Public Health, Yonsei University, 50-1 Yonsei-ro Seodaemun-gu, Seoul 03722, Korea; jejeon@yuhs.ac; 3 Department of Epidemiology and Health Promotion, Graduate School of Public Health, Yonsei University, 50-1 Yonsei-ro Seodaemun-gu, Seoul 03722, Korea; 4 Health Insurance Policy Research Institute, National Health Insurance Service, 32, Geongang-ro, Wonju-si, Gangwon-do 26464, Korea; back200@nhis.or.kr; 5 Central College, Kyonggi University, 154-42, Gwanggyosan-ro, Yeongtong-gu, Suwon-si, Gyeonggi-do 16627, Korea; kyung8545@gmail.com

**Keywords:** smoking, cardiovascular disease, young adults, trajectory, mediator

## Abstract

*Introduction*: Smoking among young adults is associated with atherosclerotic cardiovascular disease (ASCVD) in middle age. Our aim was to analyze the trajectory of smoking in young adults and analyze the effects of the trajectory group on incident ASCVD. *Methods:* This study was conducted among 60,709 young adult men aged 20–29 years who received health screening every two years from 1992–2004. Trajectory analysis was performed through smoking survey data measured 7 times during this period. ASCVD, including ischemic heart disease (IHD) and stroke events were confirmed from 2005–2015. The association between the trajectory group and ASCVD risk was analyzed using Cox proportional hazard models, controlling for covariates and mediators. *Results:* Trajectory analysis showed that smoking categorized into five groups as follows: Group 1 (28.3%), low steady; Group 2 (14.7%), lowering; Group 3 (17.3%), high steady; Group 4 (15.6%), rise and fall; and Group 5 (24.2%), very high steady. The model performance of the trajectory model (Akaike information criterion; AIC = 51,670.78) with mediators was better than the model (AIC = 51,847.85) without mediators. Group 5 showed a 49% higher risk of ASCVD than Group 1. The risk of IHD was 1.63-times higher for Group 5 and 1.31-times higher for Group 4, compared to Group 1. Compared to Group 1, Group 5 had a 1.36- and 1.58-times higher risk for total stroke and ischemic stroke, respectively. *Conclusions:* In young adult men, the multiple measured trajectory model with mediators was far more informative than one-time smoking for explaining the association with cardiovascular disease.

## 1. Introduction

Smoking is the most preventable risk factor for nearly all chronic diseases and causes of death. Smoking habits vary by individual and country but can change on a yearly basis [1]. In Korea, the rate of male smokers in 1980 was 79.3% [2], but, in 2017, it was 38.1 percent [3]. For the last 30 years in Korea, there has been a nearly 40% decrease in smoking. In this context, information gained from smoking prevalence measured at one time point is very limited. Possible applications of these measurements, however, would be a time varying exposure or a trajectory analysis of smoking [4,5,6]. Trajectory analysis of cigarette smoking is especially useful for visualizing the various smoking trends for a certain period.

Recent studies have shown that smoking in young adults is the greatest risk factor for developing middle-aged heart disease in the future [7,8,9]. This is because smoking rates are relatively high in young adults, but the prevalence of diabetes, hypertension, and dyslipidemia is low [10]. In addition, analysis of the trajectory using multiple measured smoking rates, rather than the one-time smoking rate in young adults, would better reflect the characteristics of youth smoking.

This study analyzed the trajectory of smoking in young adults for 12 years and analyzed the effects of the trajectory group on future cardiovascular outcomes in a prospective cohort during another 11 years.

## 2. Materials and Methods

### 2.1. Study Design and Participants

Prior to this year’s current insurance system, beginning in 2000, the Korean Medical Insurance Corporation (KMIC) provided health insurance for government employees and private school staff members. A total of 4,862,438 (10.7%) members of the Korean population were covered by KMIC insurance; among them, 1,297,833 were employees and 3,364,405 were dependents. All insured participants were required to participate in biennial health check-up. Among the baseline participants insured in 1992 and 1994, 94% of them were examined biennially. We established a prospective cohort for participants who were young adults aged 20–29-years-old in the KMIC and named this study as the Korean Life Course Health Study (KLCHS).

The characteristics of this study can be referenced in the previously reported Korean Cancer Prevention Study (KCPS) [2]. The KLCHS cohort included 307,041 Koreans (142,461 males and 164,580 females) who were screened by the Korean Medical Insurance Corporation in 1992 and 1994. Of these participants, 205,840 (67.0%) were registered in 1992 and 101,201 (33.0%) were registered in 1994. For this study, 60,709 male youth participated in the smoking questionnaire from 1992 to 2004 (Supplementary Figure S1).

### 2.2. Smoking History and Other Covariate Data

All participants were given the opportunity to have a medical examination every two years since 1992. The smoking history data were obtained by self-administered questionnaire. Cigarette smoking history was coded from 1–5. In the cigarette smoking variables, 1 means non-smoker, 2 means former smoker, 3 means 1–9 cigarettes per day among current smokers, 4 means 10–19 cigarettes per day, and 5 means 20 or more cigarettes per day. These smoking amount surveys were conducted every two years from 1992–2004.

Of the 142,461 male participants, 60,709 (42.6%) had their height, systolic blood pressure (SBP), fasting blood glucose (FBG), total cholesterol (TC), or body mass index (BMI) measured and were included in the study. Female participants were excluded, because of the low prevalence of smoking for females in Korea. The study proposal obtained an approval by the Institutional Review Board of Human Research, Yonsei University (4-2001-0029) and the Seoul National University (E1812/001-010). This study was a retrospective cohort using past routine laboratory data and, therefore, consent was not required.

### 2.3. Outcome

The main outcome in this study was the occurrence of atherosclerotic cardiovascular disease (ASCVD). It was classified into ischemic heart disease (IHD) (ICD-10 codes I20-I25), total stroke (ICD codes I60–I69), ischemic stroke (I63), and hemorrhagic stroke (I60–I62) [11]. Regardless of what hospital in the country study participants used, the disease ICD-10 code was reported to the National Health Insurance System. The first admission record of ASCVD in this study was defined as the onset of ASCVD event. A validity of the diagnosis of ASCVD was verified by 20 internists from the Korean Society of Cardiology in 2009 [12]. From 1994–2007, 673 cases of acute myocardial infarction were identified by private hospital records and 73% of the cases identified as myocardial infarction were valid.

### 2.4. Statistical Analysis

In the first analysis, 12-year smoking survey data, obtained every 2 years from 1992–2004, were used for the trajectory analysis, using the PROC TRAJ command in SAS. PROC TRAJ is a SAS procedure for group based modeling of longitudinal data (SAS Institute, Inc., Cary, NC, USA) [13]. Trajectory analysis uses semi-parametric group-based modeling strategies to identify potential patterns of end-to-end data. Each model represents an individual with a similar trajectory [14]. We limited the number of trajectory groups to less than five, using the Bayesian Information Criterion (BIC) to assess the best model fit [15,16]. In the second analysis, general characteristics of selected trajectory groups were compared. In a third analysis, the risk of developing heart disease, according to the selected trajectory group, was compared. At that time, the almost non-smoking group was selected as the reference group. The independent effects of the trajectory groups on ASCVD were analyzed through the Cox proportional hazard model, controlling for confounding variables. Various ASCVD models, including smoking rates measured once, smoking rate trajectory, and mediators in smoking and heart disease, were evaluated as Akaike information criterion (AIC) values, which is a well-known estimator that reflects the relative quality of statistical model for a given set of data.

## 3. Results

**Figure 1:**
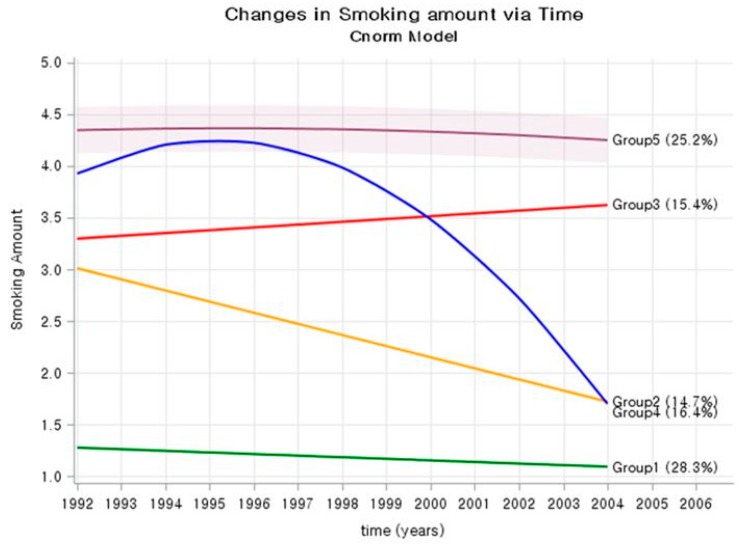
Trajectory group of smoking amounts in Korean young adult men.

The study included 8 smoking trajectory models. Among the 8 models, the model with 5 trajectory groups (1, 1, 1, 2, 2) showed the smallest BIC and was selected as the final model (Supplementary Table S1 and Supplementary Figure S1). Therefore, trajectory analysis showed that smoking categorized into five groups (Figure 1). According to the characteristics of the five groups shown in the Figure 1, the groups were named as follows: Group 1 (28.3%), low steady; Group 2 (14.7%), lowering; Group 3 (17.3%), high steady; Group 4 (15.6%), rise and fall; and Group 5 (24.2%), very high steady.

Table 1 shows general characteristics among the trajectory groups. There was no difference in age, BMI, SBP, FBG, or TC between trajectory groups in the period 1992–2004. However, the percentage of exercise was 22.1% in Group 1, 19.1% Group 2, and 20.0% Group 3, but only about 15% in Groups 4 and 5. The characteristics of Group 1 (low steady) were mostly non-smokers in 1992 and 2004. Group 2 (lowering) had the highest number of ex-smokers in 2004 (47.5%), compared to 1992. The characteristics of Group 3 (high steady) were higher in the 1–9 cigarette per day in 2004 (59.4%), than in 1992 (46.3%). Groups 4 (rise and fall) and 5 (very high steady) were mostly current smokers in 1992. Of these, the group that was still smoking in 2004 became Group 5, and the group whose smoking rate decreased sharply for any reason became Group 4.

Table 2 is a traditional model showing the relationship between smoking history at baseline and ASCVD, after controlling for confounding variables. In Model 1, current smokers had a 1.22-fold higher risk of developing ASCVD than non-smokers. In Model 2, among current smokers, smokers who smoked more than 20 cigarettes per day had a 1.52-fold greater risk of developing ASCVD.

Table 3 shows the effect of the trajectory group, estimated from 7 smoking data collections between 1992 and 2004, on ASCVD. The performance of model 1 (AIC = 51,847.85) with the trajectory group in Table 3 was similar with model 2 in Table 2 (AIC = 51,846.84) without the trajectory group. However, the performance of trajectory model 2 (AIC = 51,670.78) with mediators in Table 3 was better than the model (AIC = 51,847.85) without mediators. The trajectory model showed that the heavy smokers seemed to be divided into two trajectory groups (Group 4 and 5). In other words, the group with heavy smoking was divided into two groups: Those who maintained heavy smoking (Group 5), and those who maintained heavy smoking and then sharply decreased their smoking amount for any reason (Group 4). In Group 4, 15.6% of heavy smokers sharply decreased the amount of smoking, and there was no difference in the risk of developing ASCVD, compared to Group 1 (reference group). Group 5, on the other hand, maintained 24.2% of its heavy smokers, and their risk of ASCVD increased by 49%, compared to Group 1.

Table 4 shows the trajectory effects of smoking on IHD and stroke risk. The risk of IHD was 1.63-times higher for Group 5 and 1.31-times higher for Group 4, compared to Group 1, after adjusted for confounding variables and mediators simultaneously. Compared to Group 1, Group 5 had 1.36 and 1.58-times higher risk of total stroke and ischemic stroke, respectively. As of 1992, Group 4, which had a sudden decrease in the amount of smoking of current smokers, had a higher risk of IHD (HR = 1.31, 95% CI = 1.09–1.57), but this was not associated with the risk of stroke risk (HR = 1.01, 95% CI = 0.81–1.26).

## 4. Discussion

This study showed the trajectory of cigarette smoking and association with the risk of ASCVD, measured 7 times every two years between 1992 and 2004 in young adults. The main results were that the heavy smokers at baseline were divided into two groups: Those who maintained heavy smoking, and those who maintained heavy smoking but sharply decreased their smoking amount for any reason. As expected, reduction in the number of cigarettes smoked reduced the risk of ASCVD. The best explanatory model for the risk of ASCVD was a model with both trajectory and mediators.

In this study, Group 4 (rise and fall), which had a sudden decrease in cigarette smoking, had a higher risk of IHD, but this was not associated with an increased risk of stroke. This can be explained in two ways. First, this can be explained as an effect of smoking cigarettes on IHD before the smoking amount decreased. This is because this group showed a high smoking rate at baseline. Second, this may be due to a feeling of symptoms in the heart and a decrease in smoking. If so, this can be the result of reverse causation [17]. However, in order to determine the likelihood of reverse causation, we have excluded the initial follow-up of two years as an additional analysis. However, the results were similar.

In Group 2 (lowering), there was a steady decline in smoking between 1992 and 2004. This group actually contained many participants who had quit smoking. Although Group 2 had a high level of smoking in 1992, they quit smoking over time. As a result, there was no difference in risk of Group 1 (low steady), which also had the lowest CVD risk. This agrees with previous studies showing that smoking cessation immediately lowers the risk of heart disease [18,19].

The effect of smoking on ASCVD in this study was 1.22-fold (model 1 in Table 2), which was lower than other previous studies. This study included the subjects who participated in the smoking rate survey from 1992–2004, so we think that only the subjects who were alive until 2004 were included in the study, and our data can be explained as a kind of survival bias [20].

In this study, young adults decreased the risk of developing ASCVD to the same level as non-smokers when they steadily reduced the number of cigarettes smoked (Group 2), or when they smoked a lot of cigarettes and then steadily reduced the number of cigarettes smoked (Group 4). This meant that convincing young adult smokers to quit smoking can be a strategy to reduce the incidence of middle-aged heart disease.

A recent study on college students found smoking-related beliefs and norms recognized in individual social networks were important factors among smokers in Bangladesh [21]. Nonetheless, the reason many young people continue to smoke, and cannot stop smoking, is due to nicotine addiction. In other words, smokers are smoking cigarettes despite knowing that tobacco may not only claim many lives, but also their own [22].

The strengths of this study were that it was large-scale study of over 60,000 subjects, and that there were 7 collections of smoking data made every two years for 12 years. Through analysis of this data, we found five distinct smoking trajectories. Then, during the next 11 years, the incidences of ASCVD, including heart disease and stroke, were investigated. It is the first study to examine the effect of smoking trajectories on ASCVD events in young adults.

The biggest limitation of this study is that the amount of cigarette smoking is not a continuous variable but a categorical variable. Cigarette smoking variables were coded from 1–5. In the cigarette smoking variables, 1 means non-smoker, 2 means former smoker, 3 means 1–9 cigarettes per day among current smokers, 4 means 10–19 cigarettes per day, and 5 means more than 20 cigarettes per day. The smoking amount surveys were conducted every two years from 1992–2004. These limitations could be difficult to analyze by trajectory itself because cigarette smoking variables are categorical. Nonetheless, we used the Cnorm model of trajectory analysis [23,24]. This model was originally used for continuous variables. Fortunately, the performance of the trajectory model was excellent. More importantly, although the smoking variable is composed of categorical variables, trajectory analysis using this result shows consistent results in relation to ASCVD outcome.

In terms of generalizability, participants of KLCHS have certain limitations since they are based on government employees and private school staff members. However, since our study was to clarify the association between trajectory change of smoking patterns and ASCVD, the issue of generalizability or representativeness may not be a serious problem.

## 5. Conclusions

In conclusion, the trajectory model with mediators was far more informative to the relationship between smoking and ASCVD risk. In other words, the group who maintained steady smoking over time increased their risk of heart disease, and those who decreased smoking continuously lowered the risk of heart disease, eventually to the level of non-smokers. It is necessary to create policies to lower the smoking rate in young adults through consistent smoking education or smoker treatment.

## Figures and Tables

**Table 1:** General characteristic of study participants, according to trajectory group.

	Trajectory Group				
	Group 1 Low Steady	Group 2 Lowering	Group 3 High Steady	Group 4 Rise and Fall	Group 5 Very high Steady
*Number* (%)	17,158 (28.3)	8934 (14.7)	10,479 (17.3)	9461 (15.6)	14,679 (24.2)
Age, mean, (1992–1994)	26.9	27.0	26.9	27.1	26.9
BMI, kg/m^2^ (1992–1994)	22.3	22.3	22.2	22.6	22.7
BMI, kg/m^2^ (2002–2004)	24.1	24.2	24.1	24.7	24.5
SBP, mmHg (1992–1994)	120.2	119.4	119.2	120.1	120.0
SBP, mmHg (2002–2004)	122.6	122.6	122.5	124.2	123.7
FBG, mg/dL (1992–1994)	86.5	86.1	85.8	86.3	86.2
FBG, mg/dL (2002–2004)	90.7	91.4	91.8	93.1	93.3
TC, mg/dL (1992–1994)	174.0	173.5	174.0	176.1	176.4
TC, mg/dL (2002–2004)	192.7	193.8	194.7	199.2	197.7
Alcohol drinking (%)	67.5	83.8	83.6	84.5	85.3
Exercise (%)	22.1	19.1	20.0	15.1	13.4
Smoking status (1992)					
Non-smoker	72.6	2.4	3.8	0.7	0.3
Ex-smoker	24.7	24.8	8.5	2.7	0.7
1–9 cig/day	2.3	43.1	46.3	16.6	4.9
10–19 cig/day	0.3	23.8	36.2	50.1	49.5
≥20 cig/day	0.1	6.1	5.3	29.9	44.7
Smoking status (2004)					
Non-smoker	91.6	38.3	0.2	43.8	0.0
Ex-smoker	6.2	47.5	6.5	51.6	2.1
1–9 cig/day	1.7	11.9	28.5	3.5	2.6
10–19 cig/day	0.4	2.2	59.4	1.1	55.2
≥20 cig/day	0.1	0.1	5.3	0.0	40.1

Detailed smoking status between 1992 and 2004, attached as a supplementary table. BMI—body mass index; SBP—systolic blood pressure; FBG—fasting blood glucose; TC—serum total cholesterol; cig—cigarettes.

**Table 2:** Basic model with effect of smoking status and amount of smoking on atherosclerotic cardiovascular disease (ASCVD) events, using Cox proportional hazard model.

	HR (95% CI) in Model 1	HR (95% CI) in Model 2
Baseline variables (1992–1994)		
Age, year	1.03 (1.01–1.06)	1.03 (1.01–1.06)
Body mass index, kg/m^2^	1.09 (1.07–1.11)	1.09 (1.07–1.11)
Smoking status		
Non-smoker	1.00	1.00
Former smokers	1.13 (0.97–1.31)	1.12 (0.97–1.30)
Current smokers	1.22 (1.09–1.36)	-
1–9 cig/day	-	1.07 (0.94–1.23)
10–19 cig/day	-	1.12 (0.99–1.27)
≥20 cig/day		1.52 (1.34–1.73)
Alcohol drinking (yes)	1.00 (0.90–1.11)	1.01 (0.91–1.12)
Exercise (yes)	0.93 (0.83–1.03)	0.95 (0.85–1.05)
Systolic BP, per 10 mmHg	1.17 (1.13–1.21)	1.17 (1.13–1.21)
Serum glucose, per 10 mg/dL	0.99 (0.96–1.02)	0.99 (0.96–1.02)
Total cholesterol, per 10 mg/dL	1.06 (1.04–1.07)	1.05 (1.04–1.07)
*Number of participants*	60,709	60,709
Number ASCVD event	2392	2392
AIC	51,880.44 (DF = 9)	51846.84 (DF = 11)
Difference in AIC		33.6 (DF = 2); *p* < 0.0001

AIC—Akaike information criterion; HR—hazard ratio; CI—confidence interval; cig—cigarettes.

**Table 3:** Basic model with effect of trajectory groups and mediators on ASCVD events using Cox proportional hazard model.

	HR (95% CI) in Model 1	HR (95% CI) in Model 2
Trajectory group (1992–2004)		
Group 1 (Low steady)	1.0	1.0
Group 2 (Lowering)	1.05 (0.92–1.20)	1.05 (0.92–1.20)
Group 3 (High steady)	1.11 (0.98–1.25)	1.09 (0.97–1.23)
Group 4 (Rise and fall)	1.13 (0.99–1.29)	1.10 (0.96–1.25)
Group 5 (Very high steady)	1.49 (1.33–1.68)	1.46 (1.30–1.64)
Baseline variables (1992–1994)		
Age, year	1.04 (1.02–1.06)	1.04 (1.02–1.06)
Body mass index, kg/m^2^	1.09 (1.07–1.11)	1.07 (1.05–1.08)
Alcohol drinking	1.01 (0.91–1.12)	0.98 (0.88–1.09)
Exercise	0.94 (0.85–1.05)	0.96 (0.86–1.07)
Systolic BP, per 10 mmHg	1.17 (1.13–1.21)	1.10 (1.06–1.14)
Serum glucose, per 10 mg/dL	0.99 (0.96–1.02)	0.98 (0.95–1.01)
Total cholesterol, per 10 mg/dL	1.05 (1.04–1.07)	1.04 (1.02–1.05)
Mediators (2002–2004)		
Systolic BP, per 10 mmHg		1.19 (1.15–1.22)
Serum glucose, per 10 mg/dL		1.03 (1.01–1.04)
Total cholesterol, per 10 mg/dL		1.05 (1.02–1.04)
*Number of participants*	60,709	60,709
Number ASCVD event	2392	2392
AIC	51,847.85 (DF = 11)	51670.78 (DF = 14)
Difference in AIC		177.07 (DF = 3); *p* < 0.0001

AIC—Akaike information criterion; HR—hazard ratio; CI—confidence interval.

**Table 4:** Basic model with effect of trajectory groups and mediators on ASCVD events, using Cox proportional hazard model.

	Ischemic Heart Disease	Total Stroke	Ischemic Stroke	Hemorrhagic Stroke
	HR (95% CI)	HR (95% CI)	HR (95% CI)	HR (95% CI)
Trajectory group (1992–2004)				
Group 1 (Low steady)	1.0	1.0	1.0	1.0
Group 2 (Lowering)	1.18 (0.99–1.39)	0.99 (0.81–1.22)	1.03 (0.74–1.44)	0.79 (0.54–1.16)
Group 3 (High steady)	1.14 (0.94–1.38)	0.95 (0.75–1.21)	0.96 (0.66–1.42)	0.68 (0.43–1.09)
Group 4 (Rise and fall)	1.31 (1.09–1.57)	1.01 (0.81–1.26)	1.00 (0.69–1.44)	1.05 (0.71–1.56)
Group 5 (Very high steady)	1.63 (1.39–1.92)	1.36 (1.11–1.66)	1.58 (1.16–2.16)	1.07 (0.75–1.59)
Baseline variables (1992–1994)				
Age, year	1.05 (1.02–1.09)	1.05 (1.01–1.09)	1.07 (1.01–1.14)	1.02 (0.95–1.09)
Body mass index, kg/m^2^	1.07 (1.05–1.10)	1.03 (1.00–1.06)	1.07 (1.02–1.11)	0.96 (0.91–1.02)
Alcohol drinking	0.89 (0.77–1.02)	1.10 (0.91–1.32)	1.03 (0.77–1.38)	1.39 (0.95–2.02)
Exercise	0.92 (0.79–1.02)	0.96 (0.80–1.16)	0.94 (0.70–1.27)	1.19 (0.85–1.66)
Systolic BP, per 10 mmHg	1.09 (1.06–1.14)	1.10 (1.03–1.16)	1.09 (0.99–1.20)	1.11 (1.00–1.25)
Serum glucose, per 10 mg/dL	0.98 (0.94–1.02)	0.95 (0.91–1.01)	0.90 (0.83–0.96)	1.05 (0.96–1.14)
Total cholesterol, per 10 mg/dL	1.04 (1.02–1.06)	1.03 (1.01–1.06)	1.06 (1.02–1.10)	1.00 (0.96–1.05)
Mediators (2002–2004)				
Systolic BP, per 10 mmHg	1.10 (1.05–1.14)	1.29 (1.23–1.36)	1.20 (1.20–1.39)	1.45 (1.33–1.58)
Serum glucose, per 10 mg/dL	1.04 (1.02–1.06)	1.03 (1.00–1.06)	1.04 (1.01–1.06)	1.01 (0.96–1.07)
Total cholesterol, per 10 mg/dL	1.04 (1.02–1.06)	1.01 (0.96–1.04)	1.04 (1.01–1.07)	0.98 (0.94–1.02)
*Number of participants*	60,709	60,709	60,709	60,709
Number event	1246	795	316	222
AIC	26,970.28 (DF = 14)	17,156.18 (DF = 14)	6773.87 (DF = 14)	4789.69 (DF = 14)

AIC—Akaike information criterion; HR—hazard ratio; CI—confidence interval.

## Supplementary Materials

The following are available online at https://www.mdpi.com/1660-4601/16/12/2219/s1, Table S1: Model selection; Table S2: Changes in smoking amount by trajectory group by year, 1992–2004; Figure S1. Trajectory group of smoking amount for 8 models; Figure S2. Flow chart of participants in the study; Figure S3. Illustration of the study design.
