# Supplementary material for: Trajectory of Smoking and Incidence of Atherosclerotic Cardiovascular Disease among Korean Young Adult Men
Source: Int J Environ Res Public Health. 2019 Jun 24;16(12):2219. doi: 10.3390/ijerph16122219 (PMC6617148; doi:10.3390/ijerph16122219)
Supplement: Supplementary file 1 [file ijerph-16-02219-s001.pdf]

Supplementary Table S1. Model selection (no missing).

No.	Number of groups	Trajectory shapes	BIC
1	2	0 1	-537422.2
2	2	0 2	-534935.8
3	3	0 1 1	-508340.2
4	3	0 1 2	-507160.4
5	4	0 1 1 1	-501189.4
6	4	0 1 1 2	-500673.8
7	5	0 1 1 1 2	-485860.7
8	5	1 1 1 2 2	-485471.7

BIC: Bayesian Information Criterion.

Supplementary Table S2. Changes in smoking amount by trajectory group by year, 1992-2004.

	Trajectory group				
	Group 1 Low steady	Group 2 Lowering	Group 3 High steady	Group 4 Rise and fall	Group 5 Very high steady
N (%)	17,158 (28.3)2	10,479 (17.3)1	8,934 (14.7)3	9,461 (15.6)5	14,679 (24.2)4
Smoking status (1992)					
Never	72.6	3.8	2.4	0.7	0.3
Ex smoker	24.7	8.5	24.8	2.7	0.7
1-9 cig/day	2.3	46.3	43.1	16.6	4.9
10-19 cig/day	0.3	36.2	23.8	50.1	49.5
≥20 cig/day	0.1	5.3	6.1	29.9	44.7
Smoking status (1994)					
Never	73.8	1.5	1.6	0.2	0.1
Ex smoker	24.8	9.7	40.8	2.9	0.7
1-9 cig/day	1.1	44.9	37.6	13.7	2.7
10-19 cig/day	0.2	40.0	17.1	52.3	48.8
≥20 cig/day	0.1	4.4	2.9	30.9	47.7
Smoking status (1996)					
Never	91.5	3.7	17.0	0.4	0.3
Ex smoker	7.7	8.7	36.9	3.0	1.2
1-9 cig/day	0.7	23.7	29.9	4.5	0.7
10-19 cig/day	0.1	61.8	15.5	70.2	61.6
≥20 cig/day	0.0	2.1	0.7	21.9	36.1
Smoking status (1998)					
Never	92.1	4.4	20.2	1.0	0.7
Ex smoker	6.7	6.9	39.2	3.8	1.0
1-9 cig/day	1.0	21.4	28.6	4.4	0.7
10-19 cig/day	0.2	63.0	11.3	62.4	46.7
≥20 cig/day	0.1	4.3	0.7	28.4	51.1
Smoking status (2000)					
Never	90.9	4.9	23.5	3.5	1.1
Ex smoker	7.6	7.5	44.3	13.2	1.3
1-9 cig/day	1.1	21.5	23.5	6.8	0.9
10-19 cig/day	0.3	61.4	8.3	55.9	50.6
≥20 cig/day	0.0	4.7	0.4	20.5	46.2
Smoking status (2002)					
Never	91.2	4.7	32.9	18.6	1.3
Ex smoker	6.3	10.9	49.7	43.8	2.5
1-9 cig/day	1.7	27.8	14.0	9.1	2.9

10-19 cig/day	0.7	53.0	3.3	23.0	54.3
≥20 cig/day	0.1	3.7	0.1	5.5	39.1
Smoking status (2004)					
Never	91.6	0.2	38.3	43.8	0.0
Ex smoker	6.2	6.5	47.5	51.6	2.1
1-9 cig/day	1.7	28.5	11.9	3.5	2.6
10-19 cig/day	0.4	59.4	2.2	1.1	55.2
≥20 cig/day	0.1	5.3	0.1	0.0	40.1

<table>
<tr><th>Trajectory model</th><th>Model parameter</th></tr>
<tr><td>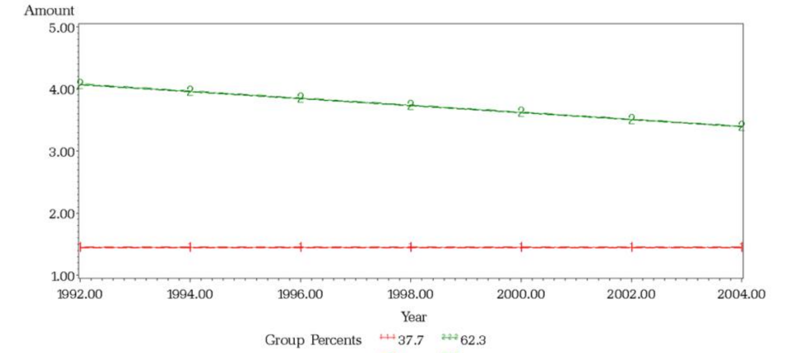
</td><td>1) order 0 1
BIC=-537422.2 (N=60711)</td></tr>
<tr><td>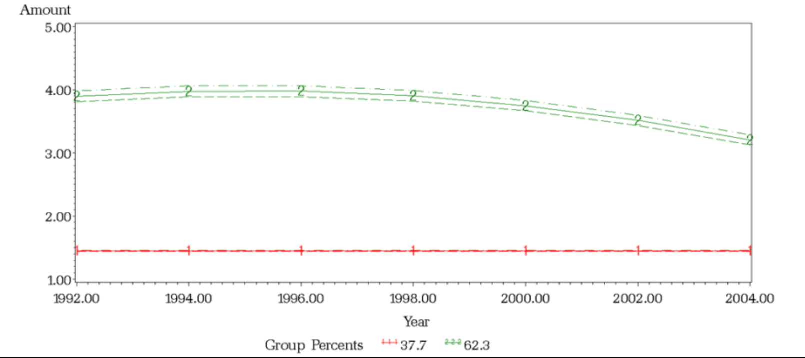
</td><td>2) order 0 2
BIC=-534935.8 (N=60711)</td></tr>
<tr><td>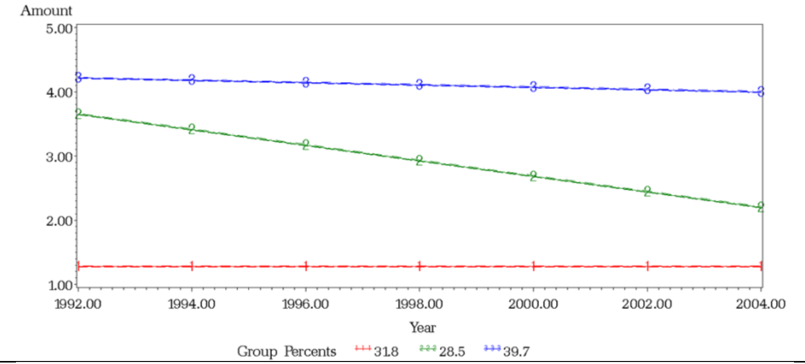
</td><td>3) order 0 1 1
BIC=-508340.2 (N=60711)</td></tr>
<tr><td>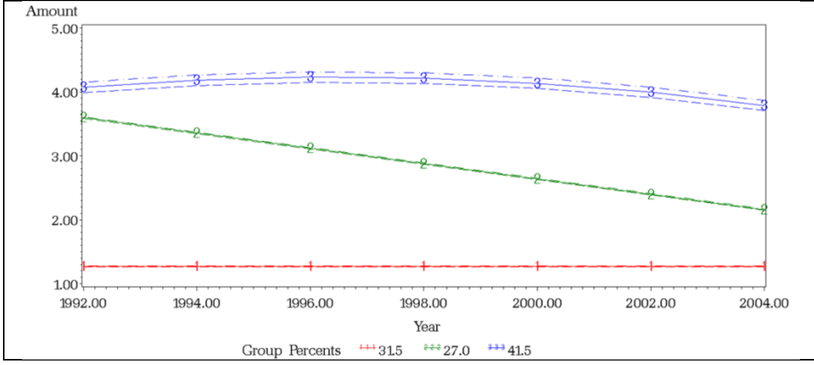
</td><td>4) order 0 1 2
BIC=-507160.4 (N=60711)</td></tr>
</table>

<table>
<tr><th>Trajectory model</th><th>Model parameter</th></tr>
<tr><td>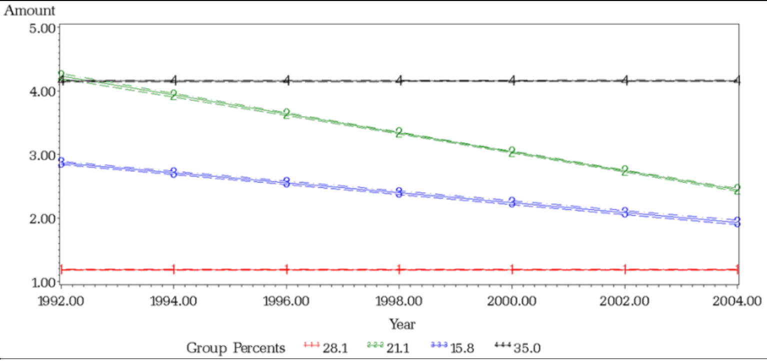
</td><td>5) order 0 1 1 1
BIC=-501189.4 (N=60711)</td></tr>
<tr><td>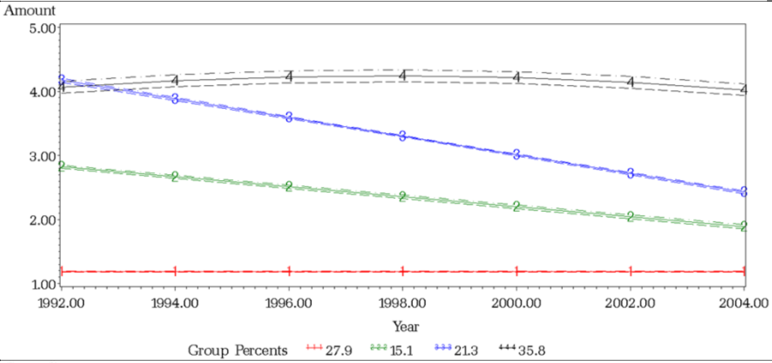
</td><td>6) order 0 1 1 2
BIC=-500673.8 (N=60711)</td></tr>
<tr><td>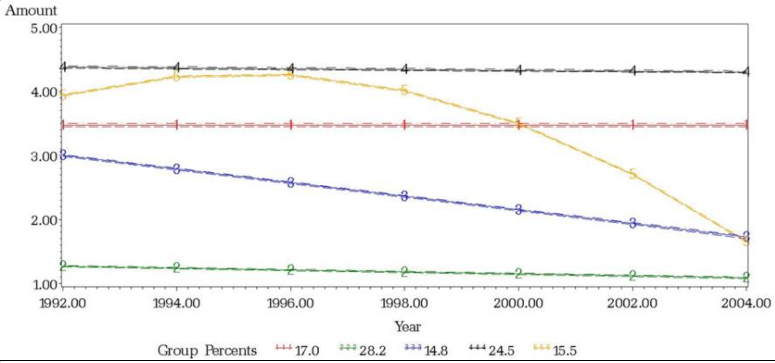
</td><td>7) order 0 1 1 1 2
BIC=-485860.7 (N=60711)</td></tr>
<tr><td>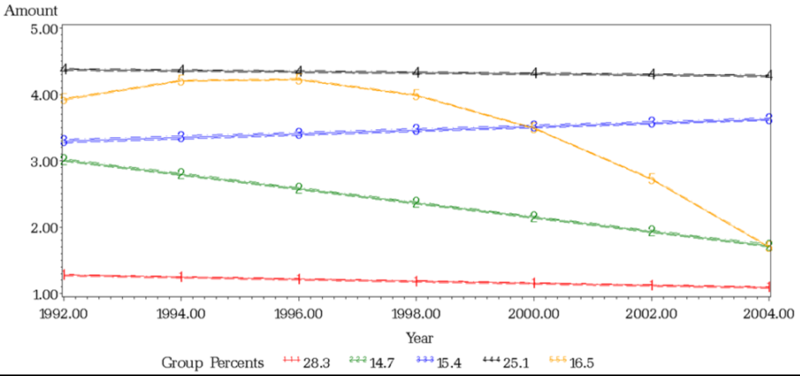
</td><td>8) order 1 1 1 1 2
BIC=-485471.7 (N=60711)</td></tr>
</table>

Supplementary Figure S1. Trajectory group of smoking amount for 8 models.

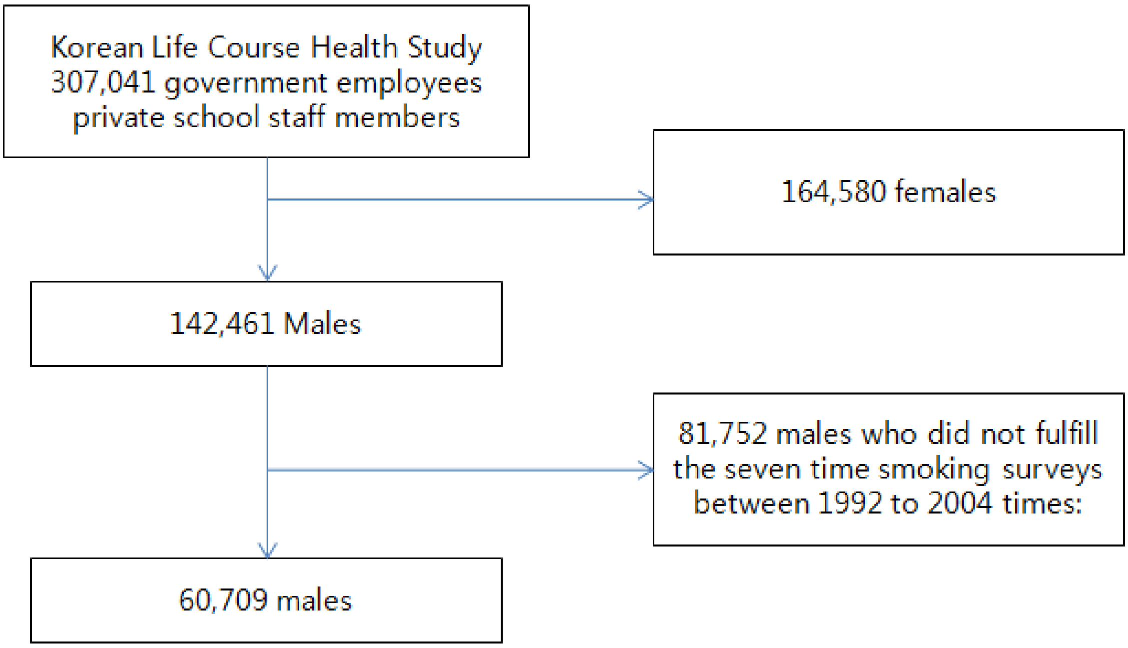

Supplementary Figure S2. Flow chart of participants in the study.

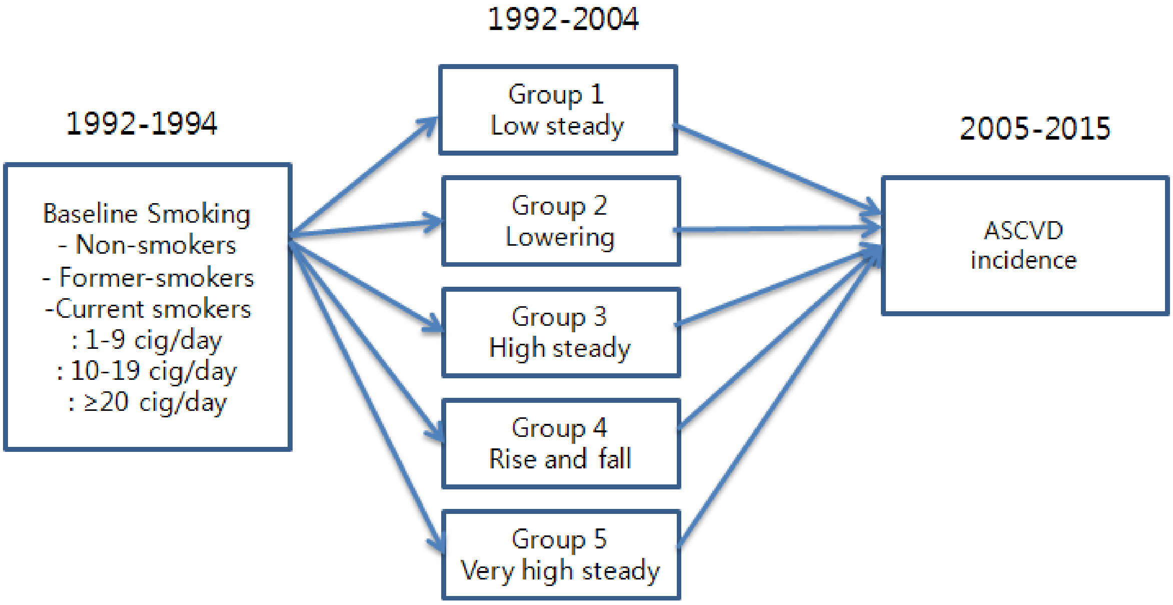

Supplementary Figure S3. Illustration of the study design.
